# Burnout and mental health issues in the psychiatry training and career pathway

**DOI:** 10.1192/j.eurpsy.2025.258

**Published:** 2025-08-26

**Authors:** E. Chumakov

**Affiliations:** Department of Psychiatry and Addiction, St Petersburg State University, St Petersburg, Russian Federation

## Abstract

**Abstract:**

Burnout and mental health issues have become increasingly prevalent among psychiatry trainees and early career psychiatrists, posing significant challenges to their well-being. This presentation explores the multifaceted relationship between psychiatry training environment and the occurrence of burnout, characterized by emotional exhaustion, depersonalization, and a diminished sense of personal accomplishment. Factors contributing to the severe burnout in psychiatry trainees include long working hours, lack of supervision, and not having regular time to rest (Jovanović N. et al., 2016). Other potential factors described include the high demands of clinical responsibilities, emotional strain from patient interactions, and inadequate support systems. Encountering possible suicidal patients and coping with patients’ violence are other factors that can have a strong influence on psychiatry trainees and early career psychiatrists’ clinical activity, especially in the absence of appropriate training in the field and supervision (Chumakov E. et al., 2022; Longo G. et al., 2023). Studies indicate that a significant proportion of psychiatry residents experience symptoms of mental distress (Pitanupong J. et al., 2024; Toni F. et al., 2024) with depression being an important source of impaired mental well-being. Furthermore, the stigma surrounding mental health within the medical community often discourages trainees from seeking help, exacerbating feelings of isolation and distress. There’s no question that poor mental health can affect not only psychiatry trainees and early career psychiatrists’ personal health but also their professional development and patient outcomes. In this presentation the implications of these issues for the psychiatry field will be discussed. There’s a growing demand for advocacy for comprehensive training programs that integrate wellness strategies, enhance supervision, and promote open conversations about mental health. By fostering a supportive environment and prioritizing self-care, the psychiatry community can mitigate the risks of burnout and mental disorders among peers, ultimately leading to a more resilient workforce and improved patient care. This presentation underscores the need to support the mental health of future psychiatrists and ensure the integrity of mental health services.

**Disclosure of Interest:**

None Declared

